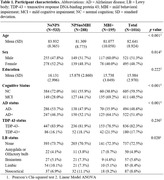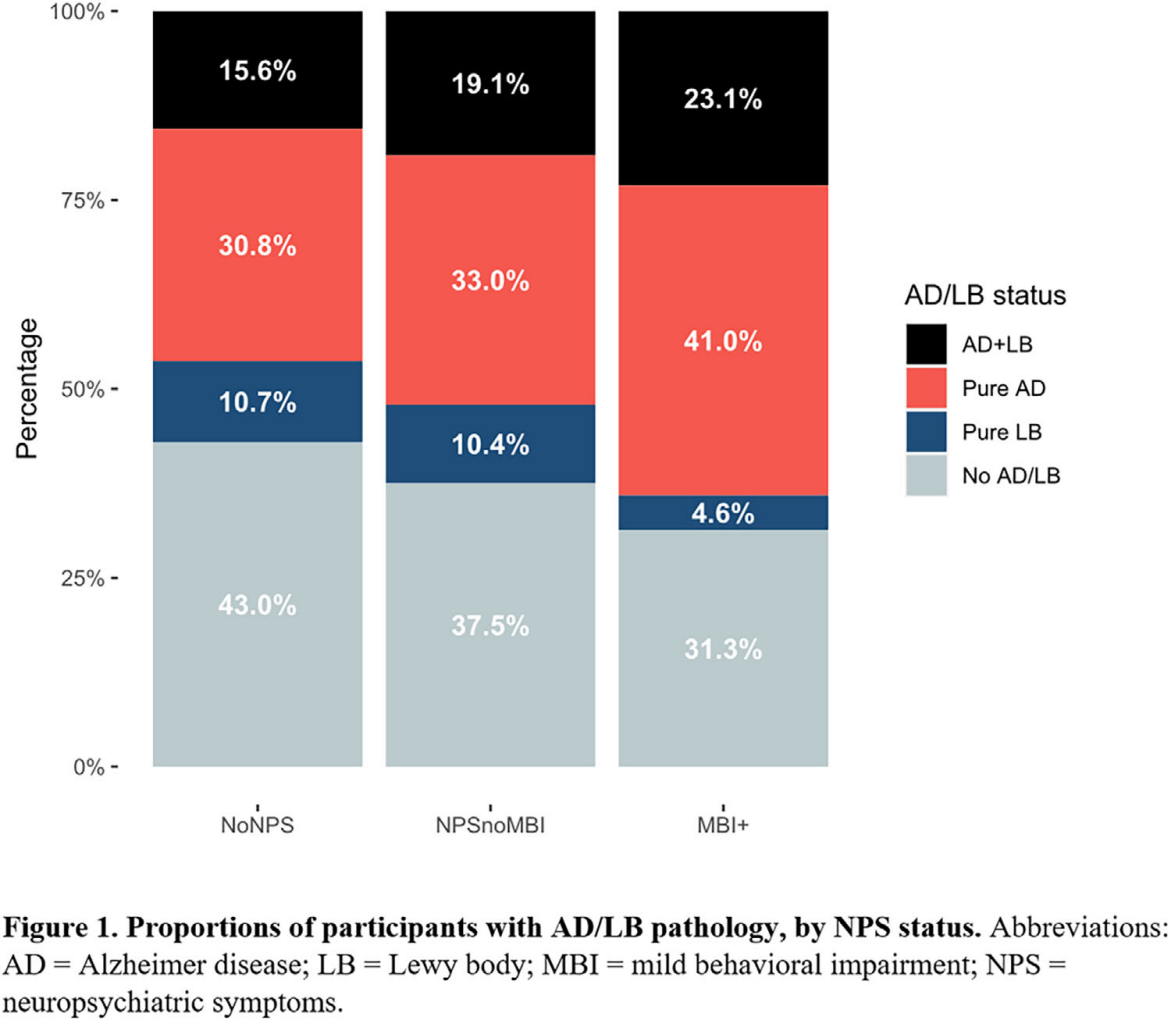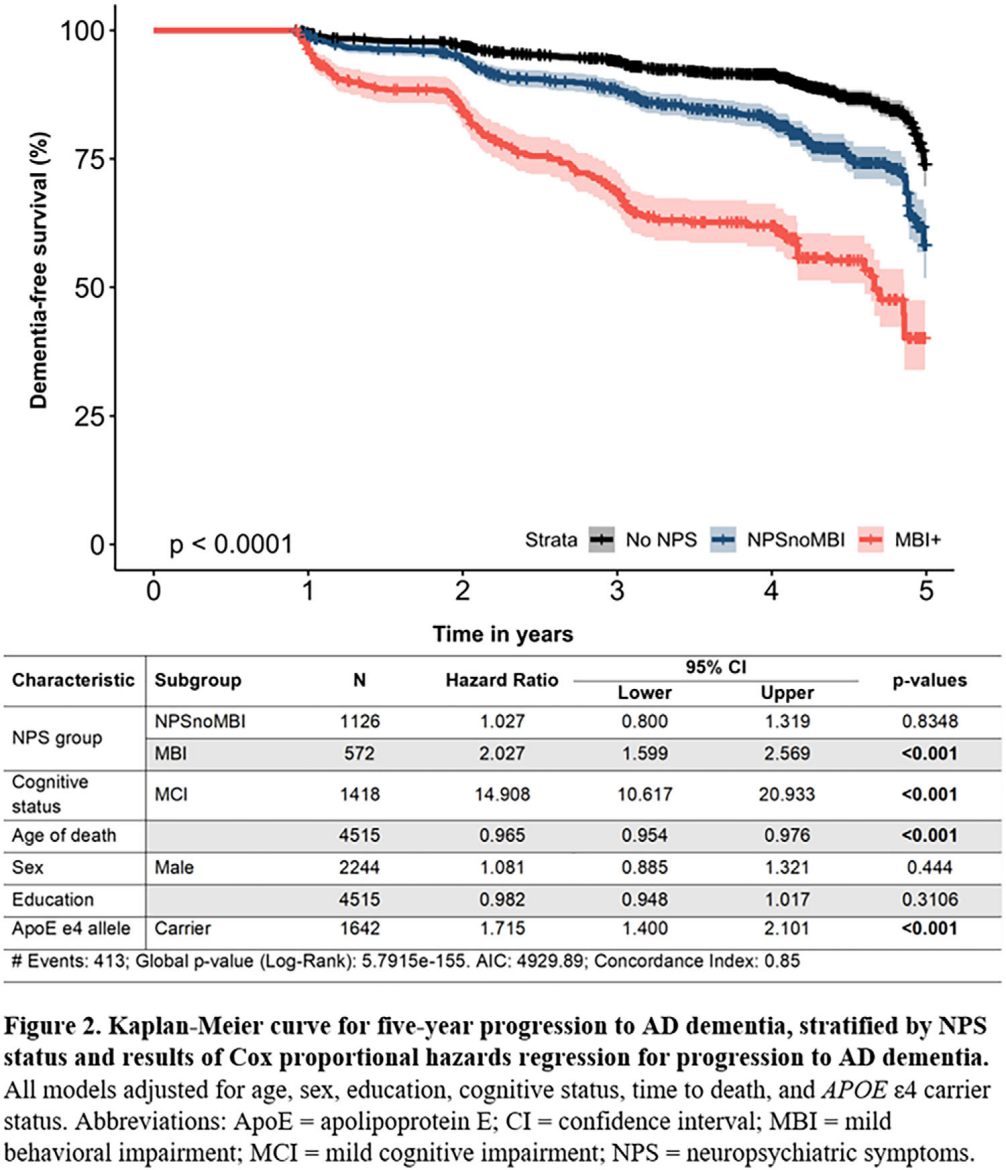# Examining the associations between Alzheimer disease, Lewy body, and TDP‐43 neuropathology with mild behavioral impairment: a clinicopathological study of older adults

**DOI:** 10.1002/alz.091539

**Published:** 2025-01-03

**Authors:** Sergio Fernandez Sharif, Dylan X. Guan, Eric E. Smith, Zahinoor Ismail

**Affiliations:** ^1^ University of Calgary, Calgary, AB Canada; ^2^ Hotchkiss Brain Institute, University of Calgary, Calgary, AB Canada

## Abstract

**Background:**

The utility of neuropsychiatric symptoms (NPS) for early detection of AD and/or other neuropathologies is not fully understood, with concerns regarding specificity. Mild behavioral impairment (MBI) is a syndrome identifying persons at risk for incident cognitive decline and dementia by leveraging risk associated with later‐life emergent and persistent NPS. Here, in dementia‐free individuals at baseline, we examined whether AD, Lewy body (LB), and/or transactive response DNA‐binding protein 43 (TDP‐43) pathology at autopsy were associated with the presence of MBI five years before death.

**Method:**

National Alzheimer’s Coordinating Center participants (n = 1016) were included. Using the Neuropsychiatric Inventory–Questionnaire, three NPS statuses were derived: MBI, NPSnoMBI, or noNPS. Proteinopathy presence/absence was determined using published guidelines. Adjusted multinomial logistic regressions modeled pathology‐NPS status associations. Adjusted Cox proportional hazards regressions modeled hazard for AD‐dementia at each NPS status level, including interaction terms with cognitive status and each co‐pathology.

**Result:**

Participant characteristics are presented in Table 1. AD+ individuals were 88.4% more likely to be MBI+ in the five years before death than AD‐ (odds ratio (OR):1.88, 95% confidence interval (CI):1.29‐2.75, p<0.01); NPSnoMBI did not differ from noNPS (OR:1.22, CI:0.90‐1.66, p = 0.20). No significant associations were seen for either co‐pathology, even in an AD‐only stratum. Among MBI progressors to dementia (n = 137), 43.8% were solely AD+ and 32.8% were mixed AD+/LB+. MBI individuals had a 2.03‐fold greater progression rate to AD‐dementia than noNPS (CI: 1.60‐2.57, p<0.01). NPSnoMBI did not differ from noNPS (OR:1.22, CI:0.90‐1.66, p = 0.20). Progression rates to AD‐dementia among MBI+ who had limbic LBs at autopsy were significantly greater than those without (HR:2.56, CI:1.09‐6.06, p<0.05). No significant interaction was noted for TDP‐43. Progression rates to dementia were higher in MCI, but the effect of MBI on progression was greater for NC (HR:3.05, CI:1.37‐6.80, p<0.01) vs. MCI (HR:1.93, CI:1.51‐2.47, p<0.01).

**Conclusion:**

These findings suggest that those with autopsy‐confirmed AD—but not other common proteinopathies—are more likely to be preceded by MBI than by NPSnoMBI or noNPS in the five years before death. Among those with MBI, the presence of LB might serve as an important co‐pathology in the development of AD dementia. By extension to postmortem diagnosis, these results reinforce the utility of MBI in AD‐related dementia prognostication.